# P-1202. Comparative Effectiveness of Host-Directed Therapies for Tuberculosis: A Systematic Review and Network Meta-Analysis

**DOI:** 10.1093/ofid/ofaf695.1395

**Published:** 2026-01-11

**Authors:** Prince darko, Neam Al-Bahdili, Kofi Berko, Zola Nladu, Dzidzo Anaglate

**Affiliations:** AU/UGA Piedmont Athens Regional Medical Center, Athens, GA; AU/UGA Piedmont Athens Regional Medical Center, Athens, GA; UNC Health Blue Ridge, North Carolina, North Carolina, North Carolina; AU/UGA Piedmont Athens Regional Medical Center, Athens, GA; AU/UGA Piedmont Athens Regional Hospital, Athens, Georgia

## Abstract

**Background:**

Host-directed therapies (HDTs) are emerging as promising adjuncts to anti-tuberculosis treatment (ATT), aiming to improve early bacteriological clearance and reduce treatment failures. This network meta-analysis (NMA) evaluated the efficacy of various HDTs in achieving negative sputum conversion at 4 and 8 weeks, using standard ATT (isoniazid, rifampin, ethambutol, pyrazinamide) as the reference

**Figure:**
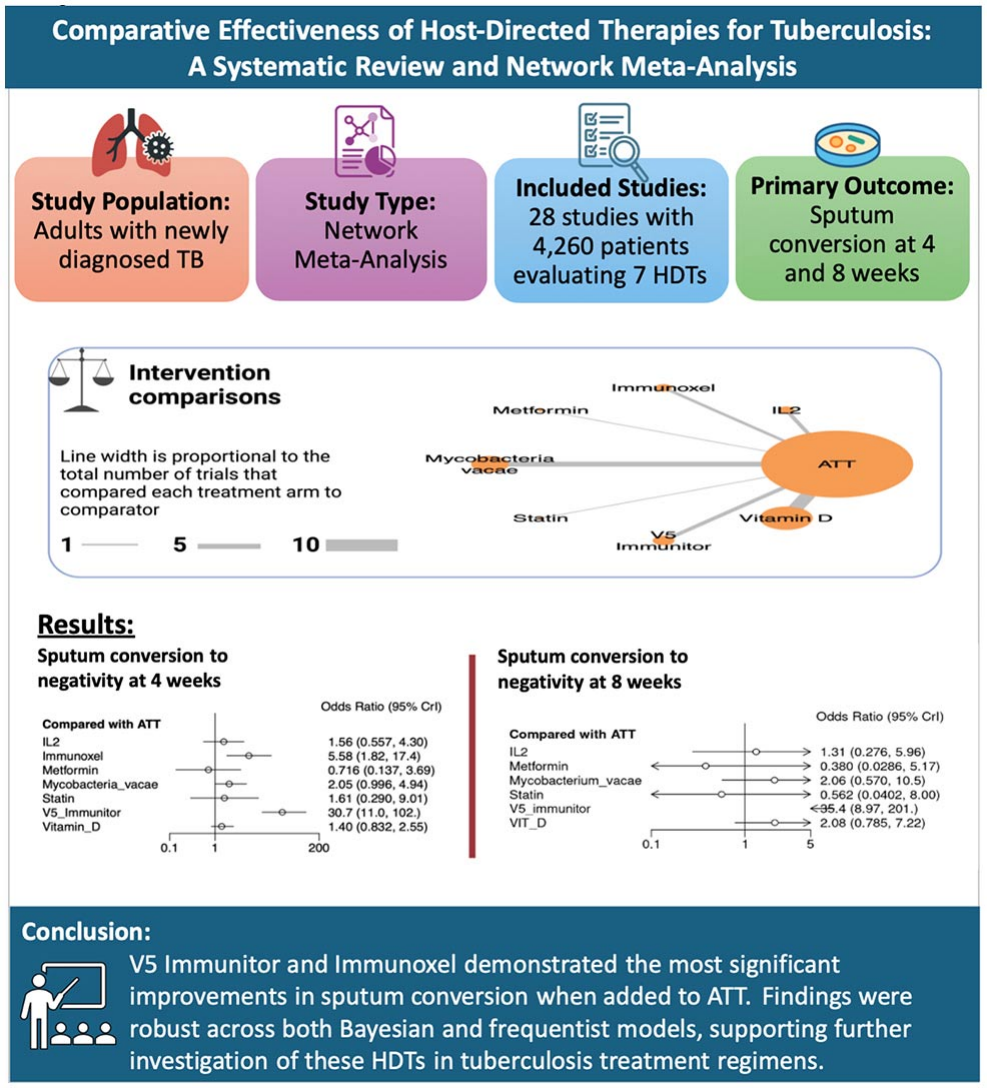
Graphical Abstract Graphical abstract showing key findings.

**Figure:**
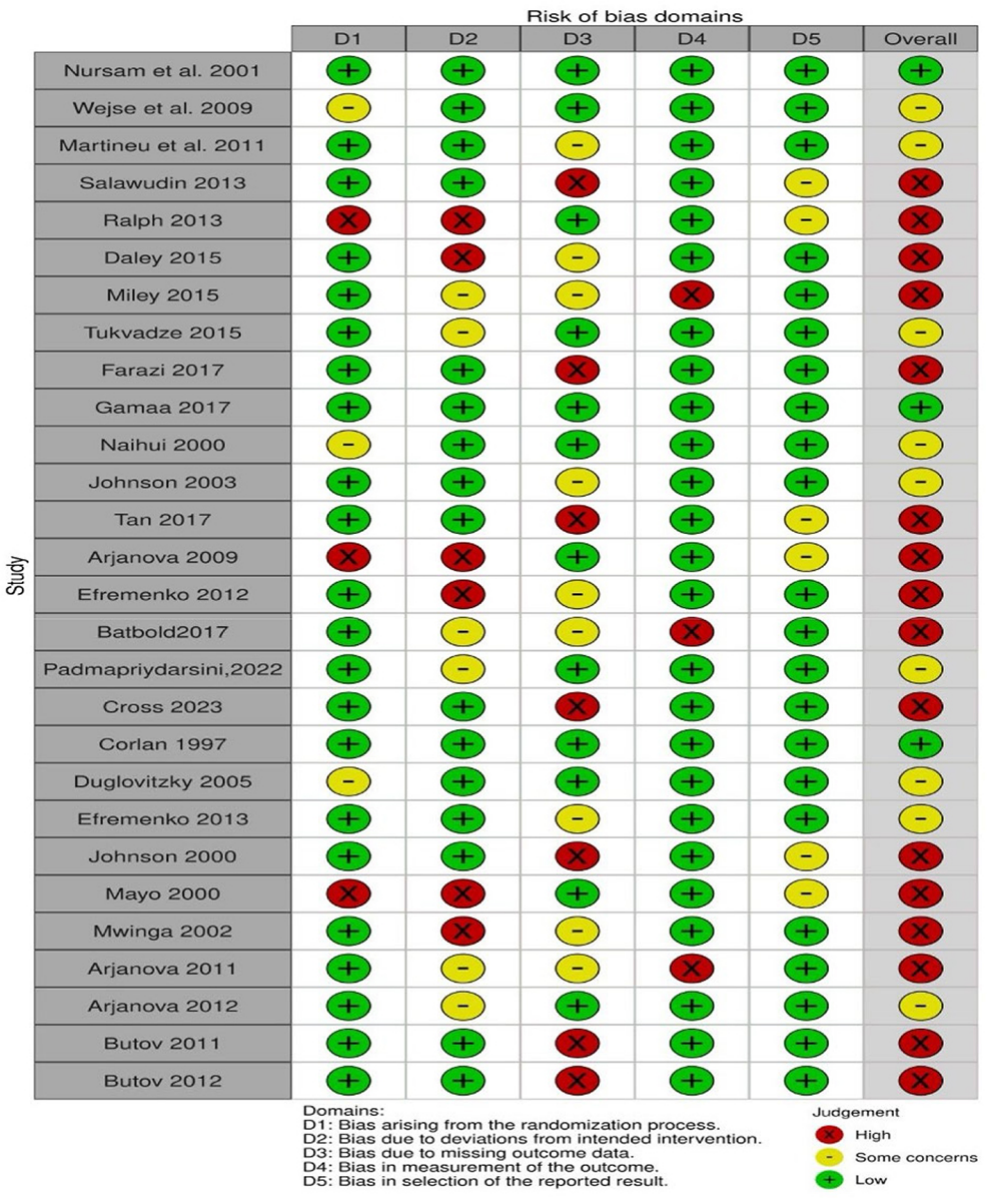
Risk of bias analysis Risk of bias

**Methods:**

A systematic review identified randomized controlled trials (RCTs) comparing HDTs—Interleukin-2 (IL-2), Immunoxel, Metformin, Mycobacterium vaccae, Statins, V5 Immunitor, and Vitamin D—plus ATT versus ATT alone. A Bayesian random-effects NMA using a binomial likelihood with logit link estimated odds ratios (ORs) and 95% credible intervals (CrIs). A frequentist NMA was conducted for comparison. Outcomes were sputum conversion at 4 and 8 weeks

**Figure:**
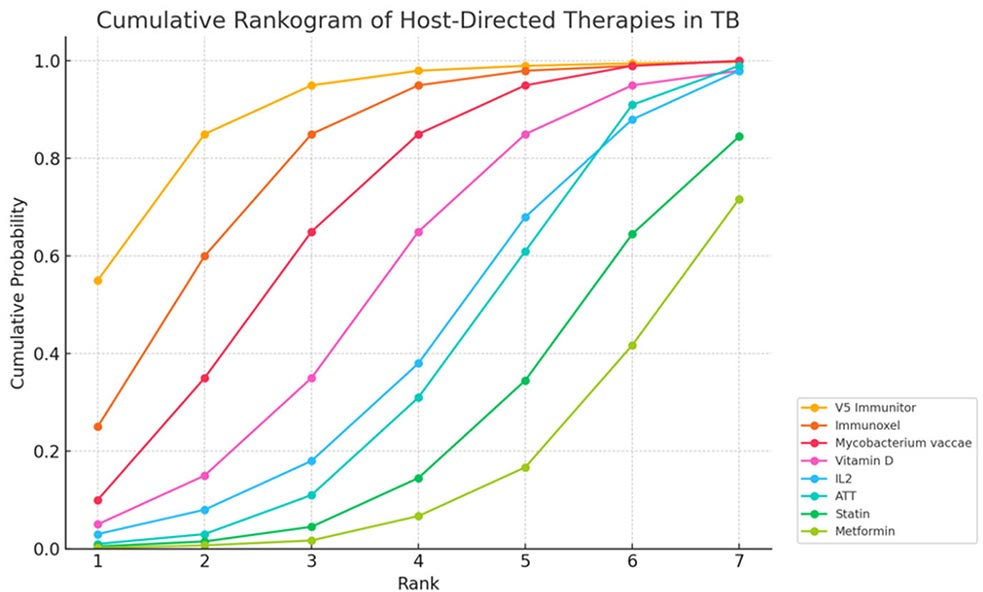
Cumulative rankogram of host directed therapies in Tuberculosis Radiogram showing pictorial representation of various treatment arms

**Figure:**
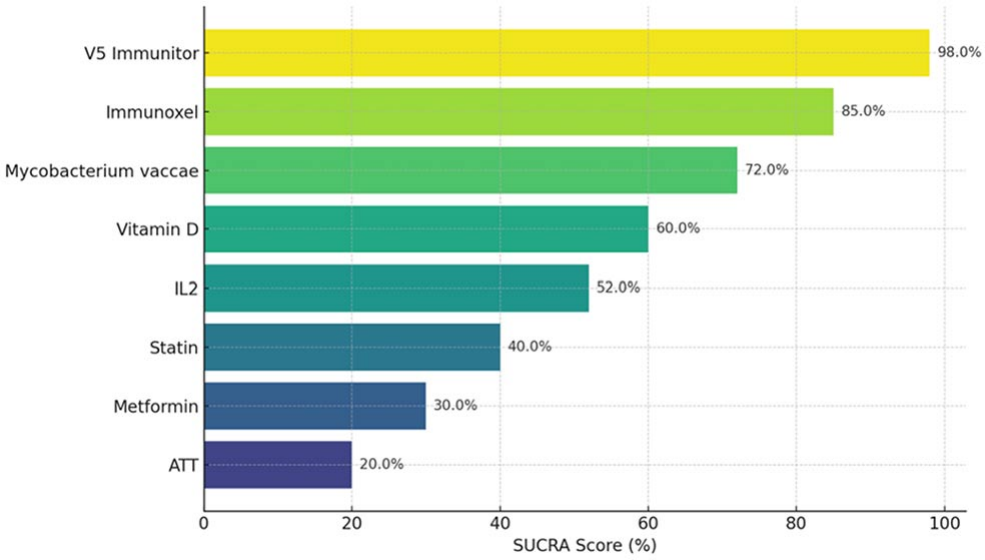
Graph showing SUCRA scores of Host directed therapies in Tuberculosis management Graph showing sucre scores of the various HDTs used in the studies analyzed.

**Results:**

Twenty-eight RCTs (4,260 patients) were included. At 4 weeks, V5 Immunitor (OR 30.7; CrI 11.0–102), Immunoxel (OR 5.68; CrI 1.82–17.4), and Mycobacterium vaccae (OR 2.05; CrI 0.996–4.94) ranked highest. IL-2, Vitamin D, and Statins showed modest, non-significant effects; Metformin had lower efficacy (OR 0.716; CrI 0.137–3.69). At 8 weeks, V5 Immunitor remained top (OR 35.4; CrI 8.89–201), followed by Vitamin D and M. vaccae. Frequentist models confirmed large effects for V5 Immunitor and Immunoxel for sputum conversion to negative at 4 weeks. Heterogeneity was moderate.

**Conclusion:**

V5 Immunitor and Immunoxel significantly improved early sputum conversion when added to ATT. Findings were consistent across Bayesian and frequentist models, supporting potential inclussion of HDTs in tuberculosis therapy.

**Disclosures:**

All Authors: No reported disclosures

